# Supplementary material for: A methodology for the design of experiments in computational intelligence with multiple regression models
Source: PeerJ. 2016 Dec 1;4:e2721. doi: 10.7717/peerj.2721 (PMC5136129; doi:10.7717/peerj.2721)
Supplement: Data S1 — Datailed results from UC Irvine Machine Learning Repository (Housing, Machine CPU, Wine Quality, Automobile and Parkinson) and the 3 Use Cases (Protein Corona, Gajewicz Metal Oxides and Aquatic Toxicity) [file peerj-04-2721-s001.zip › 6_UseCases/2_GajewiczMetalOxides/RFRFE.details.csv.repeatedcv.split1.pdf]

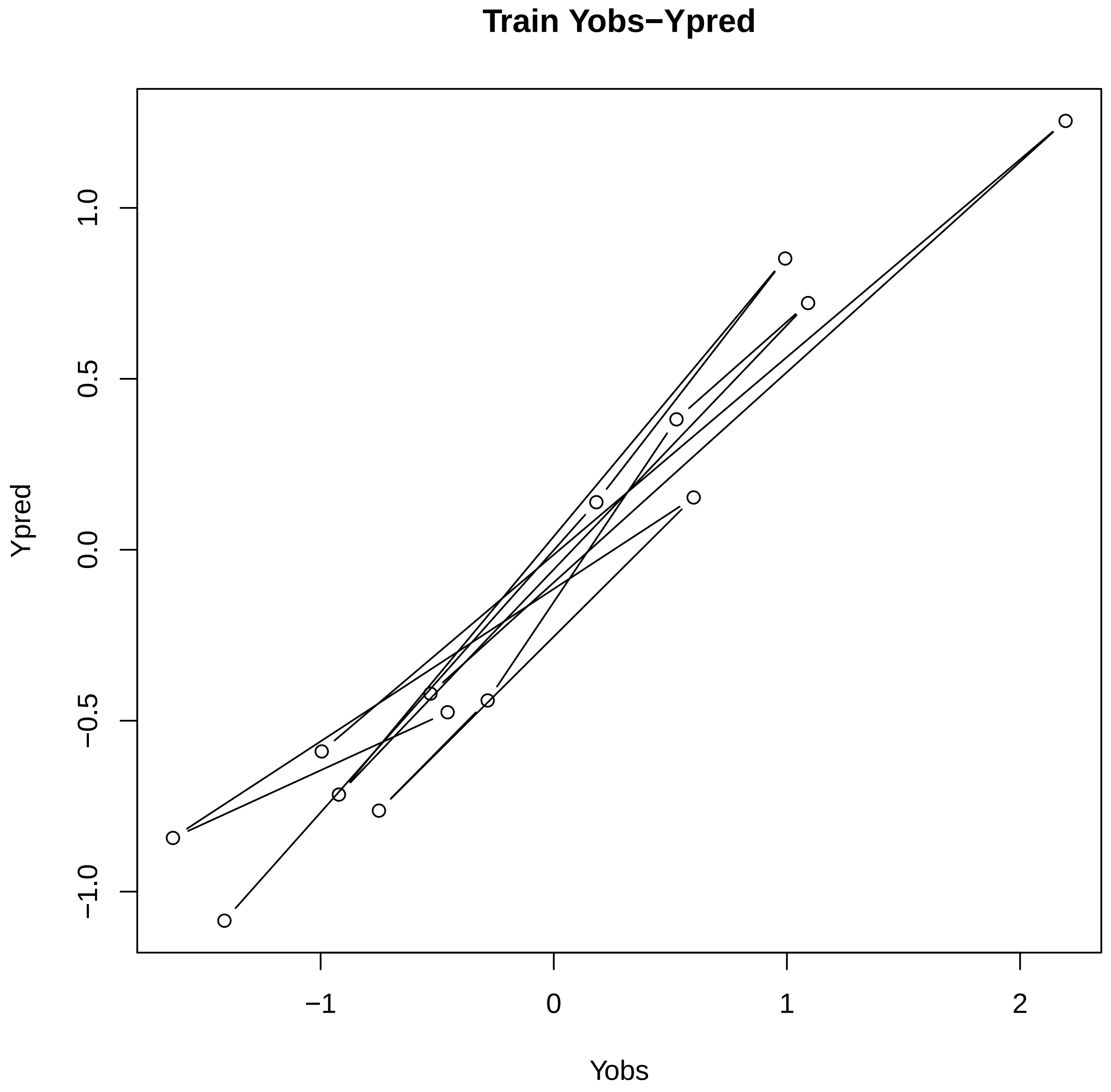

Train Yobs−Ypred
Ypred
Yobs
1.0
0.5
0.0
−0.5
−1.0
−1
0
1
2

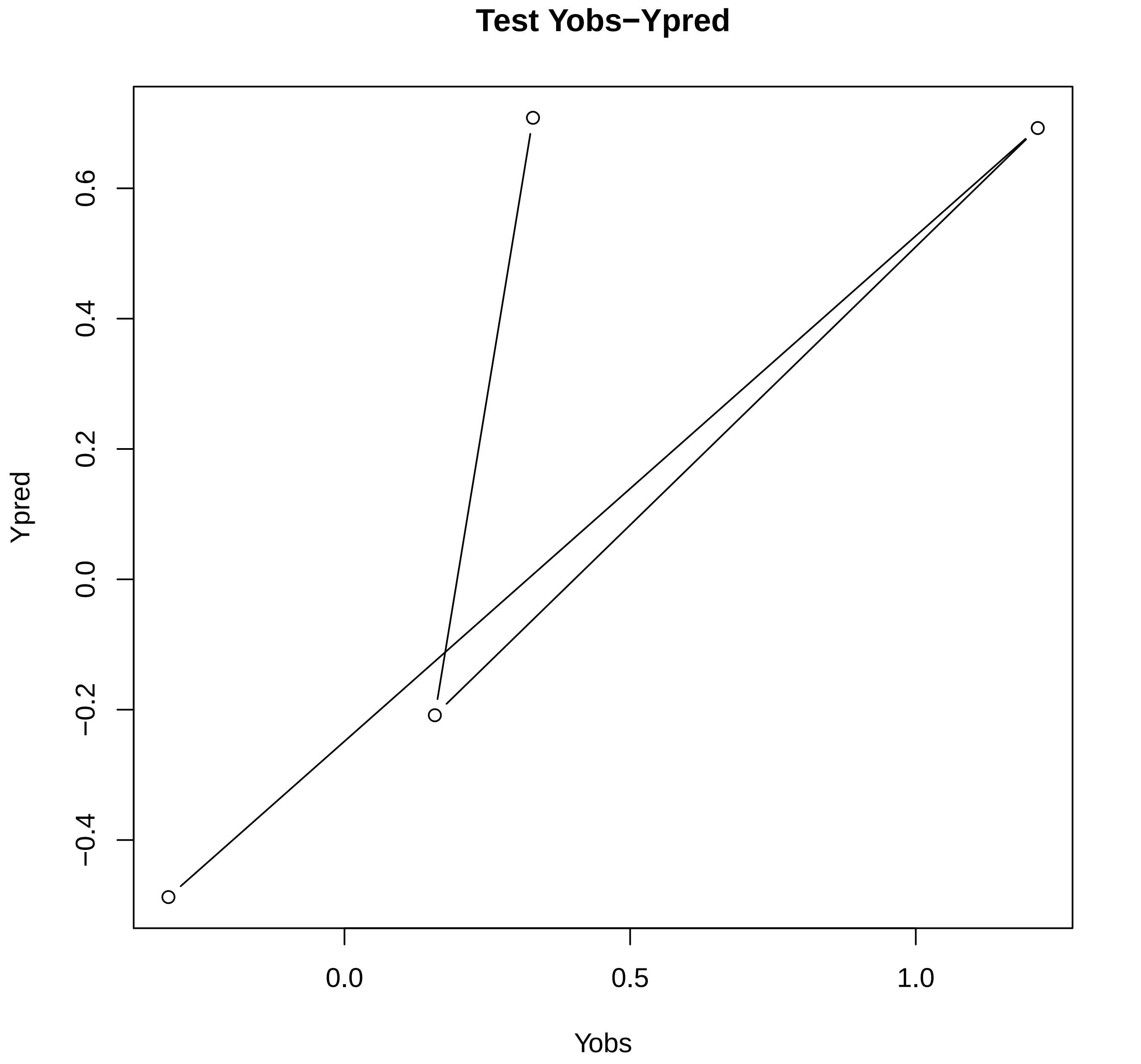

Test Yobs−Ypred
Ypred
0.6
0.4
0.2
0.0
−0.2
−0.4
0.0
0.5
1.0
Yobs

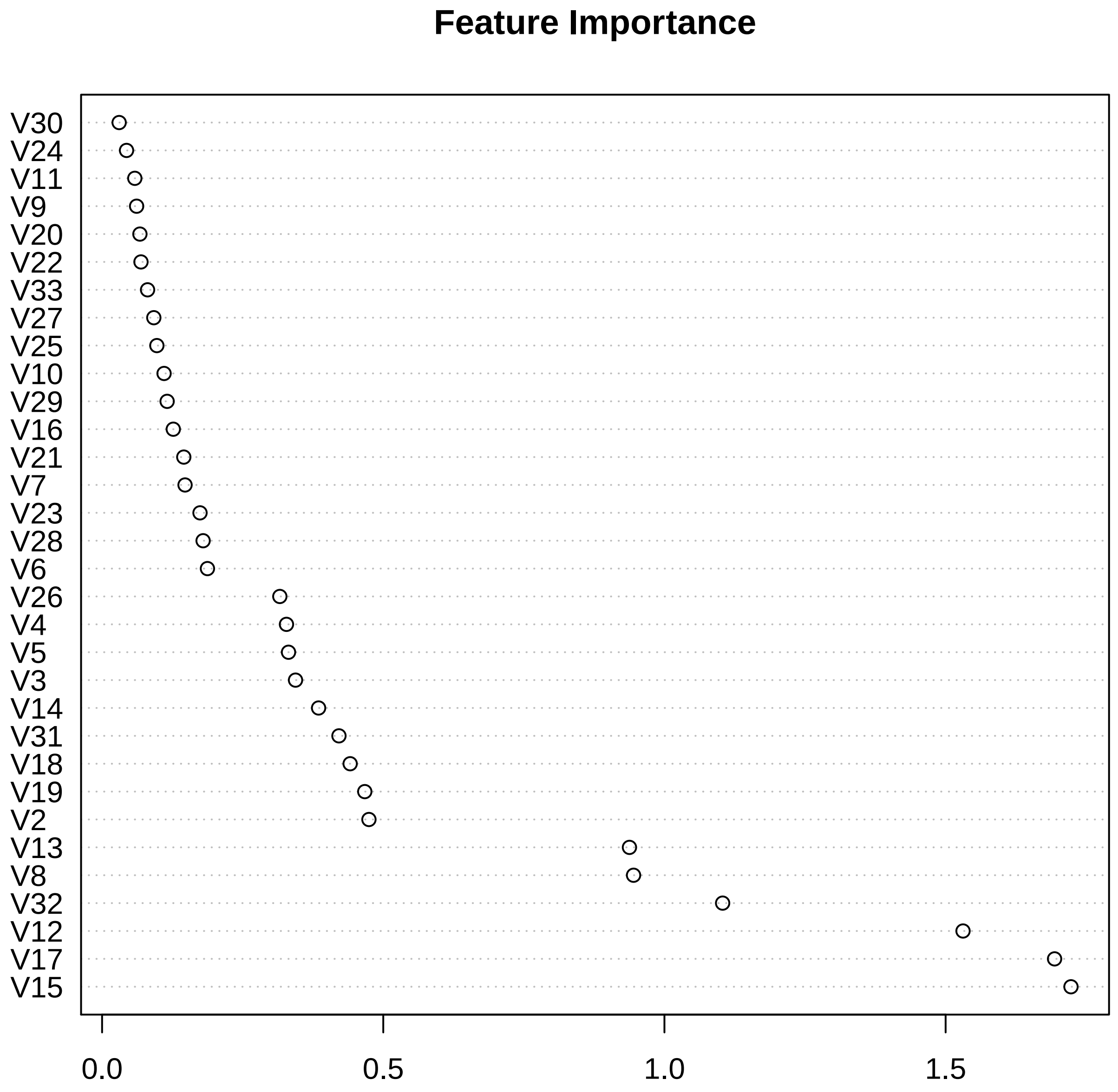
Feature Importance
V30
V24
V11
V9
V20
V22
V33
V27
V25
V10
V29
V16
V21
V7
V23
V28
V6
V26
V4
V5
V3
V14
V31
V18
V19
V2
V13
V8
V32
V12
V17
V15
0.0
0.5
1.0
1.5

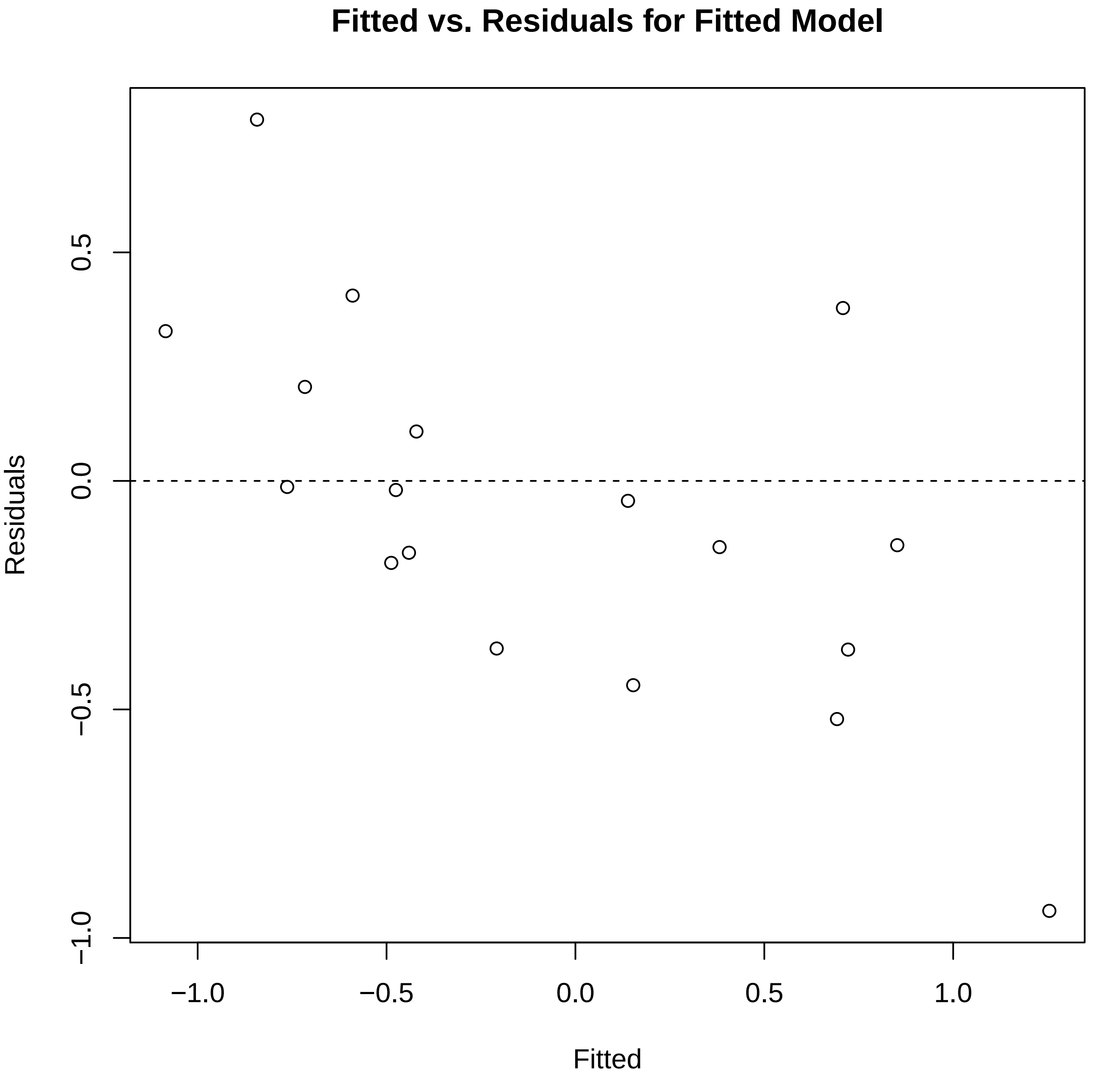
Fitted vs. Residuals for Fitted Model
Residuals
Fitted
0.5
0.0
−0.5
−1.0
−1.0
−0.5
0.0
0.5
1.0
